# Comment on ‘Impact of Cachexia and First‐Line Systemic Therapy for Previously Untreated Advanced Non‐Small Cell Lung Cancer: NEJ050A’ by Miura et al.

**DOI:** 10.1002/jcsm.13657

**Published:** 2024-11-06

**Authors:** Wei‐Zhen Tang, Wei‐Ze Xu, Tai‐Hang Liu

**Affiliations:** ^1^ Department of Bioinformatics, School of Basic Medical Sciences Chongqing Medical University Chongqing China

After a thorough analysis of the latest research findings by Keita Miura and colleagues, we affirm their conclusion that approximately one‐third of untreated patients with advanced non–small cell lung cancer (NSCLC) exhibit cachexia before first‐line systemic treatment, with different treatment methods having a significant impact on these patients' appetite‐related quality of life, weight changes and survival [1]. However, the study may have overlooked some key issues that could affect the accuracy and interpretation of the results.

Firstly, the study failed to exclude patients with severe gastrointestinal diseases, active malignancies other than NSCLC, symptomatic brain metastases or those requiring high‐dose steroid treatment. These factors are important variables to consider when assessing the impact of cachexia as they could significantly affect the patient's nutritional status, quality of life and response to treatment [2, 3]. If these patients were not excluded, these conditions could have caused a significant confounding effect on the study's conclusions. For instance, severe gastrointestinal diseases could directly impact appetite and food absorption, similar to the appetite decline and weight loss observed in cachexia. Still, they may not be directly related to cancer or its treatment. This could lead to inaccurate assessments of cachexia as non–cancer‐related weight loss could be mistakenly attributed to cancer‐related cachexia. Similarly, malignancies other than NSCLC could increase systemic inflammatory responses, promoting the development of cachexia, while symptomatic brain metastases requiring high‐dose steroids could induce or exacerbate changes in weight and appetite, thus confounding the assessment of cachexia. Additionally, other side effects of steroid treatment, such as muscle wasting and immunosuppression, could be confounded with the physiological changes of cachexia, further complicating the interpretation of treatment effects and patient prognosis [4]. Therefore, these unexcluded conditions could mask the true effects of cachexia treatment, making it difficult to accurately assess the impact of cachexia on patient quality of life and survival.

Secondly, the study did not record and adjust for pulmonary comorbidities that could significantly affect cachexia, such as baseline chronic obstructive pulmonary disease (COPD) and emphysema. These chronic lung diseases not only affect lung function but could also impact the overall health status of patients, including nutritional status and quality of life, especially in patients with advanced NSCLC. If these key baseline conditions were not recorded and adjusted for, they could introduce significant confounders into the study, affecting the assessment and interpretation of cachexia. COPD and emphysema could lead to breathlessness, reduced activity tolerance and decreased quality of life, symptoms similar to those of cachexia, including weight loss, reduced appetite and muscle wasting (Sepulveda‐Loyola et al. 2020). Therefore, failing to consider these chronic lung diseases could overestimate or underestimate the incidence and severity of cachexia, thus affecting the understanding of the relationship between cachexia and treatment outcomes [5, 6]. Additionally, COPD and emphysema could affect a patient's tolerance to treatments and choices of treatment, including chemotherapy, targeted therapy and immunotherapy. For example, severe COPD could limit the ability of patients to receive certain chemotherapeutic drugs as these could further impair lung function. This limitation in treatment choice could affect a patient's treatment response and survival, potentially misattributing treatment effects to the presence of cachexia without proper adjustment for these factors. Furthermore, specifics about completed chemotherapy cycles were not recorded. Previous studies have found that increased severity of cachexia is associated with fewer completed cycles of chemotherapy, suggesting that cachexia could affect a patient's tolerance to chemotherapy and the continuity of treatment. Without recording the number of chemotherapy cycles, this could limit our comprehensive understanding of how cachexia affects the treatment response and prognosis of NSCLC patients. Since the number of chemotherapy cycles is a key indicator of treatment continuity and completeness, the lack of this data could lead to an inability to accurately assess the impact of cachexia on a patient's ability to complete chemotherapy [7]. Cachexia could lead to more treatment‐related side effects, such as weight loss, reduced appetite and fatigue, which could affect a patient's ability to complete all planned cycles of chemotherapy. Therefore, not recording the number of chemotherapy cycles could obscure the potential negative impact of cachexia on treatment continuity and effectiveness. Recording the number of chemotherapy cycles is crucial for assessing treatment effects, cumulative toxicity, patient tolerance to treatment and changes in quality of life. In the treatment of advanced NSCLC, the number of chemotherapy cycles could be closely related to the efficacy of the drugs, disease control rate (DCR), progression‐free survival (PFS) and overall survival (OS). Due to the lack of this data in the documents, we cannot accurately assess the impact of chemotherapy dose density on patient prognosis nor can we understand whether patients need to adjust their treatment plans due to tolerance issues.

Lastly, the study did not focus on the information about the intensity and duration of physical exercise of the participants. Physical exercise is thought to reduce systemic inflammation and promote muscle anabolic metabolism, which is particularly important for cancer patients, as cancer cachexia syndrome often accompanies muscle wasting and systemic inflammation. Physical exercise could mitigate these negative effects by reducing inflammation and promoting muscle health, potentially improving quality of life and survival rates [8]. Additionally, the study did not record the specific use of drugs like Anamorelin or corticosteroids and progestogens for the treatment of cachexia. These drugs are thought to enhance appetite and promote anabolic metabolism, potentially effective for improving symptoms of cachexia in cancer patients, especially Anamorelin as a ghrelin receptor agonist, which could have a positive effect on stimulating appetite and muscle synthesis [9, 10]. While corticosteroids can increase appetite in the short term, long‐term use could lead to side effects. Since the study did not record the use of these therapeutic drugs, it is not possible to assess their potential impact on cachexia symptoms, appetite changes, muscle mass and patient survival.

## Conflicts of Interest

The authors declare no conflicts of interest.
